# Corrosion Mitigation in Molten Salt Environments

**DOI:** 10.3390/ma17030581

**Published:** 2024-01-25

**Authors:** Sylvie Delpech, Charly Carrière, Alexandre Chmakoff, Laure Martinelli, Davide Rodrigues, Céline Cannes

**Affiliations:** 1 IJCLab, CNRS/IN2P3, Université Paris-Saclay, 91405 Orsay, France; charly.carriere@ijclab.in2p3.fr (C.C.); alexandre.chmakoff@cea.fr (A.C.); rodrigues@ijclab.in2p3.fr (D.R.); celine.cannes@ijclab.in2p3.fr (C.C.); 2 Service de Recherche en Corrosion et Comportement des Matériaux, Commissariat à l’Energie Atomique et aux Energies Alternatives (CEA), Université Paris-Saclay, 91190 Gif-sur-Yvette, France; laure.martinelli@cea.fr

**Keywords:** halide molten salts, corrosion, thermodynamic, redox control

## Abstract

The aim of this paper is to present methods for corrosion mitigation in molten salt environments. The corrosion of structural materials depends directly on the redox potential of the salt. When the redox potential of the salt is higher than the standard potentials of the elements constituting the structural materials, corrosion occurs. If the reverse is true, no corrosion is observed. Herein, a methodology for calculating the theoretical potential of a molten salt is provided and compared with experimental measurements. Three ways to mitigate corrosion by modifying the salt redox potential are proposed: (i) using a soluble/soluble redox system; (ii) using a potentiostatic method; and (iii) using an amphoteric compound such as UCl_3_, TiCl_2_, or TiCl_3_. Immersion tests were conducted under the above conditions to validate the methodology.

## 1. Introduction

Corrosion mitigation in a molten salt environment is a complex challenge because molten salts can be highly corrosive due to their ability to chemically react with a wide range of materials. Molten salts are often used in various industrial processes, such as advanced energy applications (molten salt reactors (MSRs) for nuclear power generation and concentrated solar power plants) [1]; pyrochemical processes for nuclear fuel treatment and chemical processes such as the carbonitriding of steels [2]; the synthesis of fluorine gas [3], aluminum [4], and sodium [5]; and the separation of Hf/Zr [3]. In molten salt environments, in contrast to aqueous solutions, passivating layers cannot be stabilized with a strong protective effect. The corrosion in halide salts seems to proceed via active dissolution without the presence of an oxide layer. Depending on the amount of oxide, the metal degrades due to its oxidation with the formation of (i) a soluble chloride salt or (ii) an oxide that is subsequently dissolved [6]. Raiman and Lee [7] aggregated data and analyzed corrosion in fluoride and chloride salts as a function of several parameters: salt purity, temperature, material, container material, and experimental method. The strongest correlation was observed between corrosion and salt purity. However, in all cases, the corrosion of the alloys (Fe- or Ni-based) was observed. Generally speaking, and as it is clearly shown in the reference [7], papers on corrosion in molten salts present very different corrosion results for the same alloy or metal depending on the authors. In our opinion, this is due to the non repeatability of the medium in terms of redox and oxo-acidity between authors. The origin of the salts, their purity, that of the gases used, and the crucible materials nature can induce differences in the reactivity between the environment and the materials. As part of this publication, we focused our study on the influence of redox potential on the corrosion of materials.

The aim of this paper is to present a thermodynamic approach to corrosion in molten salts in order to define methods for its reduction or inhibition by controlling the salt redox potential; illustrative results are also provided. Three strategies are presented in this paper:

- The addition of a redox system;
- The potentiostatic control of the material potential;
- The addition of an amphoteric compound.

Though the approach and results presented herein are applicable elsewhere, this paper is focused on molten salt reactor (MSR) development. The concept of the molten salt reactor (MSR) was developed in the 1960s by the Oak Ridge National Laboratory (ORNL), USA. This type of reactor is well adapted to the thorium fuel cycle. When the MSRE (molten salt reactor experiment) was designed in 1960, the primary objective was a safe, reliable, and maintainable reactor. The level of success achieved was described by Haubenreich [8]. The molten salt chosen for the first MSR design was a fluoride salt suitable for the Th-U nuclear fuel cycle. In France, the nuclear industry has operated with the U-Pu fuel cycle since its initiation. For several decades, France has been looking for a breeder reactor in order to minimize the consumption of fissile material. Two options are currently being studied: the Fast Sodium Reactor (SFR) and the MSR with a fast neutron spectrum.

To produce a chain reaction with a high breeding ratio, the U/Pu nuclear fuel cycle requires a fast neutron spectrum; this is difficult to achieve in fluoride salts because of the small size of fluoride ions, which thermalizes the neutrons in the high energy domain [9]. Therefore, in order to increase the breeding ratio, the nature of the salt had to be modified, and a chloride salt based on NaCl containing fertile and fissile materials UCl_3_ and PuCl_3_, respectively, was chosen [10]. The second reason why chloride salt was chosen is the possibility of treating used salt in French reprocessing plants (La Hague) because chloride salts are very soluble in an aqueous medium, unlike fluoride salts.

## 2. Experimental Methods

Thermodynamic calculations were realized using the HSC Chemistry 6.0 database of pure compounds.

**Figure 1:**
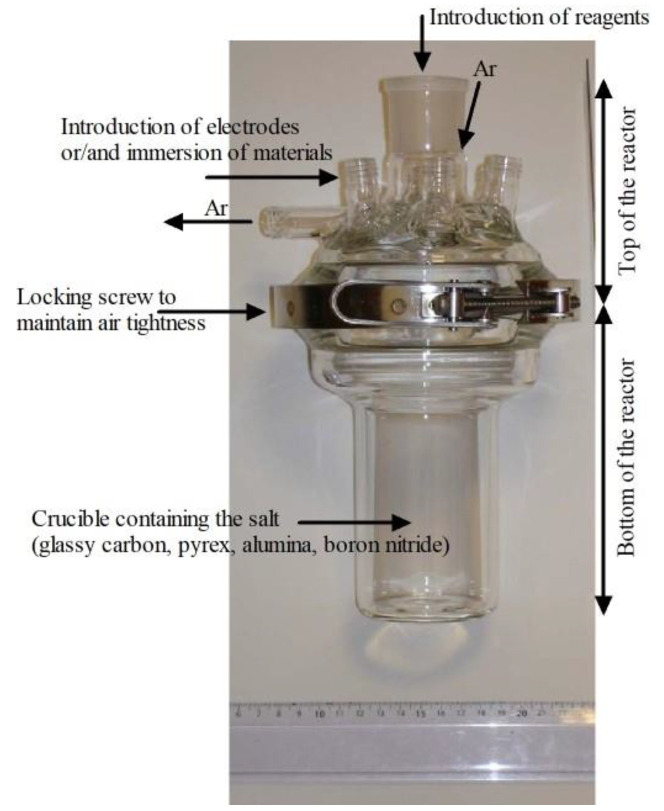
Reactor used for molten salt studies.

The experimental measurements were conducted in a reactor comprising two parts (Figure 1). The holes in the top of the reactor were used to introduce the electrodes, immerse the materials, and ensure gas flow in the cell. Generally, the gas was an inert gas such as Ar. The molten salts were stored, weighed, mixed, and introduced into the crucible in an inert glove box (H_2_O and O_2_ content < 5 ppm) and then transferred to the cell under a fume hood. Airtightness was maintained using a locking screw.

The electrochemical cell was then placed in a tubular furnace (80 cm diameter) connected to a regulation monitor provided by ERALY (Fontenay-le-Fleury, France). Electrochemical measurements were performed using a PAR 263A potentiostat/galvanostat coupled with a PC. The salt was purified by keeping the cell under vacuum for 24 h at 300 °C. Then, the mixture was melted by increasing the temperature under vacuum. Once the salt had melted, a flow of argon (Ar AlphaGaz 1, supplied by Air Liquide, Paris, France) was maintained throughout the experiment. The airtightness of the top of the cell was ensured by introducing electrodes through SVL caps.

The working electrodes were plates of the studied materials (Hastelloy C276 (Ni57/Mo17/Cr16/Fe5/W4/Mn1 in wt%) and AISI 304L (Fe/Cr18/Ni10 in wt%)) or pure metal wires 1 mm in diameter (Ti, Cr, Ni, Cu, Fe, Al, Mg, Zr, Ta, Au, Pd, Pt, Ru, Mo, and W) provided by Goodfellow (Huntingdon, UK). The counter electrode was a 3.05 mm diameter graphite rod provided by ThermoScientific (99.9995% purity, Waltham, MA, USA). A AgCl/Ag redox system was chosen as the reference electrode because of its reversible charge transfer and its high stability in chloride molten salts. For the electrode preparation, a Pyrex glass tube was filled with molten LiCl-KCl salt containing 0.75 mol.kg^−1^ AgCl, and a Ag wire (provided by Goodfellow) was dipped inside. A junction potential of 2 mV was measured for this Pyrex glass compartment. The potential of the AgCl/Ag electrode was −1.033 V/(Cl_2_/Cl^−^) [11].

Salts containing LiCl, KCl, ThF_4_, and LiF are discussed in this paper. The halide salts (anhydrous, with 99% purity) were provided by Sigma-Aldrich, St Quentin Fallavier, France. ThF_4_ was provided by Solvay (Brussels, Belgium).

## 3. Thermodynamic Approach to Corrosion

**Figure 2:**
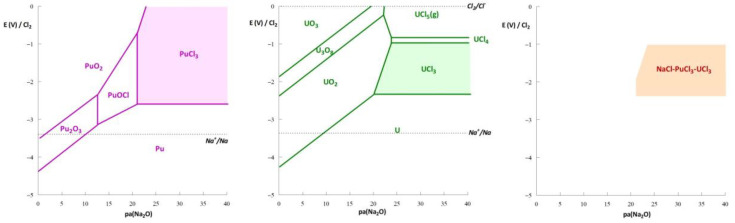
Thermodynamic diagrams of uranium (**left**) and plutonium (**middle**) in NaCl-based chloride media and the salt NaCl-UCl_3_-PuCl_3_ (80-10-10 mol%) (**right**) at 600 °C. Mole fractions of U and Pu were taken as 10 mol% for these examples.

Thermodynamic diagrams, or potential–pH (E-pH) diagrams, similar to those presented by Pourbaix in aqueous solution [12], were constructed to define the stability domains of different forms (ions, complex, precipitate, metal) of a chemical element on an E–acidity graph. In molten halide salts, the acidity is often defined by the activity of oxide ions (O^2−^), which is called oxo-acidity [13]. A typical methodology used to construct these diagrams is presented in [14]. In the case of molten salt reactors using a sodium-based chloride salt, the oxide particle generally considered for thermodynamic calculations (based on a pure compound database) is Na_2_O. Therefore, the diagrams are presented as E-pa(Na_2_O) graphs, with pa(Na_2_O) being the cologarithm of Na_2_O (pa(Na_2_O) = −loga(Na_2_O)). The reference chosen for the potential scale was the system Cl_2_(1atm)/Cl^−^ (a = 1). Figure 2 presents diagrams constructed for uranium and plutonium in a chloride salt and the stability domain of the ternary system. These diagrams are simplified and do not consider the formation of oxides containing the cation Na^+^. Such forms are stable in the basic domain of the diagram and are not relevant to this paper.

The orange domain shown in Figure 2 corresponds to the stability of the molten mixture NaCl-UCl_3-_PuCl_3_. This is the only zone wherein all the elements are soluble and under chloride form. If the system shifts to another domain (after introducing O_2_, H_2_O, HCl, Cl_2_, etc.), an oxide or oxychloride is precipitated, or the fissile/fertile elements are reduced to metals. Therefore, the stability domain of a nuclear fuel salt is defined by

−2.2V < E < −1 V/(Cl_2_/Cl^−^) and pa(Na_2_O) > 25 (depending on the potential).

**Figure 3:**
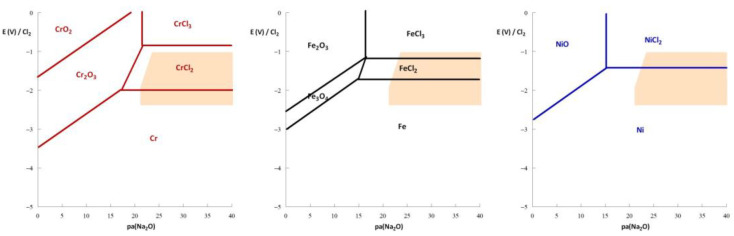
Thermodynamic diagrams of Cr (**left**), Fe (**middle**), and Ni (**right**) in NaCl-based molten salt at 600 °C. The orange domain corresponds to the stability domain of the NaCl-UCl_3_-PuCl_3_ (80-10-10 mol%) mixture. a(MCl_2_) = 10^−6^ (M = Cr, Fe, and Ni).

Figure 3 presents thermodynamic diagrams of the pure metals Cr, Fe, and Ni, which are the main constituents of the alloys used in the nuclear industry. Under the conditions of nuclear operation (orange zone), the formation of an oxide layer that could protect the structural materials is incompatible with the nuclear fuel salt stability domain. In the operating range of a reactor, Cr, Fe, and Ni exist under two states: soluble or metallic. In contrast to aqueous solutions [12], in molten salts, metallic elements have immunity domains. Therefore, corrosion can be mitigated by controlling the redox potential of the materials or molten salt.

This is true for other applications. The presence of impurities [15] such as oxides in the salt increases corrosion. An increase in oxides decreases the pa(Na_2_O) value, which, as shown by the thermodynamic diagrams, decreases the oxidation potential of the metals and makes them more easily oxidable. This is expected, considering the complexation of metals with oxides. Unfortunately, in molten salts, the formation of an oxide layer brings no protection due to its dissolution [16,17].

**Figure 4:**
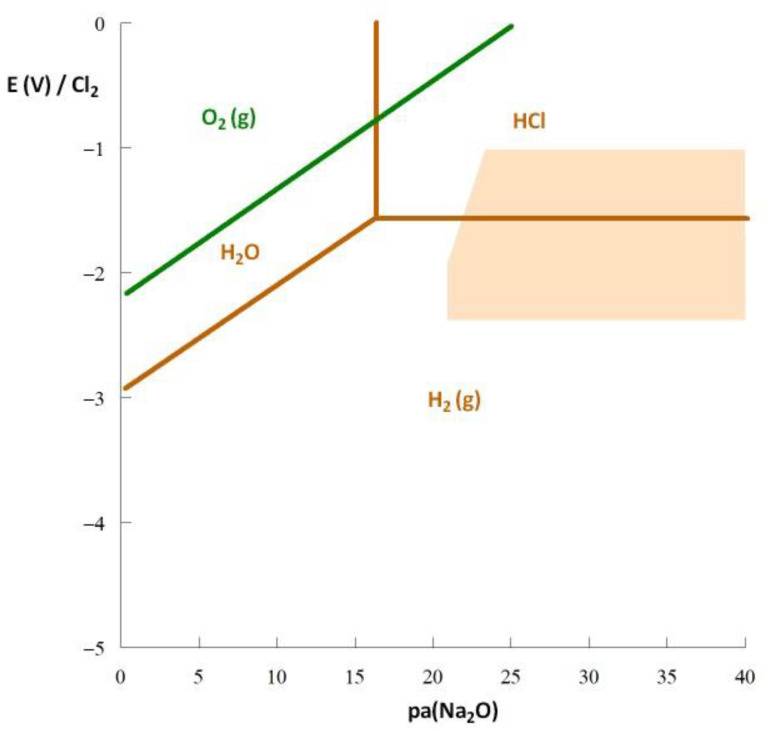
Thermodynamic diagram of hydrogen and oxygen in a NaCl-based chloride salt at 600 °C. The orange domain corresponds to the stability domain of the NaCl-UCl_3_-PuCl_3_ (80-10-10 mol%) mixture. The partial pressure of the gases is 10^−3^ atm.

The role of water and oxygen was studied using thermodynamic calculations. The stability domains of O_2_, H_2_O, HCl, and H_2_ were calculated, and their limits are presented in Figure 4. The figure shows that O_2_ is not stable in the stability domain of the fuel salt (defined by the orange zone). Under the conditions of oxo-acidity required to stabilize the salt in soluble conditions, O_2_ reacts with the chloride ions to produce Cl_2_ gas as follows:

O_2_ (g) + 2NaCl → Na_2_O + Cl_2_ (g) (1)

Water is not stable in this context either. When H_2_O is introduced to the salt, it reacts to produce an oxide and HCl gas as follows:

H_2_O (g) + 2NaCl → Na_2_O + 2HCl (g) (2)

Reactions (1) and (2) reveal the production of HCl (g) and Cl_2_ (g) in the salt, which are strong oxidants. Therefore, the addition of water or oxygen increases the oxidizing power of the salt.

The potential can be controlled by a gas mixture of HCl/H_2_. By decreasing the P(HCl)/P(H_2_) ratio, the potential can be decreased to reach the immunity domain of the elements Cr, Fe, and Ni. The same reasoning applies to fluoride salts and HF/H_2_ gas mixtures are traditionally used to purify the fluoride salts in MSRs [18].

HCl can be formed due to traces of water in the cover gas or the salts. For example, the use of MgCl_2_-based salts is characterized by the production of HCl during the salt melting process [19]. Corrosion increases with HCl formation but can be mitigated by introducing Mg metal into the salt [20,21]. In these conditions, the redox potential of the salt decreases from −1.36 V/Cl_2_ to −1.93 V/Cl_2_, corresponding to the immunity domain of Cr, Fe, and Ni. The Mg metal reduces HCl by the following reaction:

Mg + 2HCl → MgCl_2_ + H_2_ (g) (3)

The salt potential increases after adding NaOH to the salt, which can be explained by the formation of HCl as follows:

NaOH + MgCl_2_ → MgOHCl + NaCl (4)

MgOHCl → MgO + HCl(5)

Therefore, the redox potential of the salt increases due to the formation of HCl.

In all cases, the corrosion of alloys containing chromium in a NaCl-MgCl_2_ salt is essentially due to a chemical reaction between Cr and MgCl_2_ producing MgCr_2_O_4_ [19], possibly taking the following form:

2Cr + 4MgCl_2_ + 4Na_2_O → MgCr_2_O_4_ + 8NaCl + 3Mg ΔG = −890.798 kJ

The thermodynamic diagrams show (i) no compatibility between the high purity of the salt (low concentrations of oxides) and the “theoretical” passivity domain of the structural elements (corresponding to the formation of an oxide compound) and (ii) compatibility between the immunity domain of the structural elements and the stability domain of the nuclear fuel salt. Therefore, to inhibit the corrosion of these alloys, the redox potential of the salt must be kept in the immunity domain of the structural elements. The thermodynamic diagrams also show that the lower the oxo-acidity, the smaller the redox potential corresponding to the immunity domain of the metallic element and, therefore, the more difficult it is to achieve.

## 4. Salt Redox Potential Calculation and Measurement

### 4.1. Theoretical Salt Redox Potential

The theoretical determination of a salt redox potential is challenging. Several authors have related the redox potential of a salt to the Fermi level and conduction bands [22,23] and have compared the electroactivity ranges of various Fermi levels and valence and conduction bands. However, the Fermi level in this model does not consider the differences in the electrons involved in the anodic and cathodic processes (the limits of the electroactivity range) and is placed midway between the valence and conduction bands, conflicting with the experimental measurements.

Thermodynamics could also be used to calculate the theoretical potential of a salt by considering the salt decomposition equilibrium. Two examples are given below.

- The case of LiCl-KCl (59–41 mol%) at 500 °C

The anodic and cathodic limits of LiCl-KCl are, respectively, the formation of chlorine gas and the reduction of Li^+^, according to the following reactions:

2Cl^−^ → Cl_2_(g) + 2e (6)

LiCl + e → Li + Cl^−^ (7)

The Nersnt relations associated with these reactions are

(8) $$E_{Cl2/Cl}=E{{}^{\circ}}_{Cl2/Cl}+\frac{2.3RT}{2F}\log\frac{a\left(Cl_{2}\right)}{a{\left(Cl^{-}\right)}^{2}}$$

(9) $$E_{LiCl/Li}=E{{}^{\circ}}_{LiCl/Li}+\frac{2.3RT}{F}\log\frac{a\left(LiCl\right)}{a(Li)a(Cl^{-})}$$

where R is the ideal gas constant (J/K/mol); T is the temperature (K); F is the Faraday constant (C/mol); a(M) represents the activity of element M; and E_M_ and E°_M_ are the potential and standard potential, respectively, of the redox system of M.

The salt decomposition equilibrium reaction is

LiCl → Li + 1/2Cl_2_(g) (10)

Therefore, at equilibrium (assuming that the concentration and activities are equal):

a(Cl_2_) = ½ a(Li) (11)

The potential of the salt E_LiKCl_ is given by the Nernst Relations (8) and (9). Therefore,

(12) $$E_{LiKCl}=E_{Cl2/Cl}=E_{LiCl/Li}$$

and the sum of E_Cl2/Cl_ and E_LiCl/Li_ leads to

(13) $$3E_{LiKCl}=2E{{}^{\circ}}_{Cl2/Cl}+E{{}^{\circ}}_{LiCl/Li}+\frac{2.3RT}{F}\log\frac{a(Cl_{2})a(LiCl)}{a{\left(Cl^{-}\right)}^{3}a(Li)}$$

Combining Relations (11) and (13), we have

(14) 3ELiKCl=2E°Cl2/Cl+E°LiCl/Li+2.3RTFlog12 a(LiCl)a(Cl−)3

(15) ELiKCl=2E°Cl2/Cl+E°LiCl/Li3+2.3RT3Flog12 a(LiCl)a(Cl−)3

E°_LiCl/Li_ can be determined using the thermochemical data of pure compounds, i.e., −3.57 V/Cl_2_ at 500 °C. E°C_l2/Cl_ is equal to 0, as this is the reference potential. The activity of LiCl was determined by Lumdsen [24] as equal to 0.38. We consider LiCl-KCl to be totally dissociated, and therefore, the activity of the chloride ions a(Cl^−^) is equal to 1. Using these values, we can determine a theoretical value for the LiCl-KCl salt potential:

E_LiKCl_ = −1.22V/Cl_2_. (16)

This value can be compared with the open-circuit potential (OCP) measured experimentally on an inert electrode of tungsten in the molten salt, which is close to −1.25 V/Cl_2_ [25,26].

- The case of NaCl-MgCl_2_ (55–45 mol%) at 500 °C

Using the same methodology, we can determine the thermodynamic redox potential of NaCl-MgCl_2_ (0.55–0.45 mol%) at 500 °C, E_MgNaCl_. In this case, the cathodic limit corresponds to the reduction of MgCl_2_ to Mg metal. The relation of E_NaMgCl_ can be established as follows:

(17) $$E_{NaMgCl}=\frac{E{{}^{\circ}}_{Cl2/Cl}+E{{}^{\circ}}_{MgCl2/Mg}}{2}+\frac{2.3RT}{4F}\log\frac{\;a(MgCl_{2})}{a{\left(Cl^{-}\right)}^{4}}$$

considering that the activity of MgCl_2_ is equal to its mole fraction (0.45) and that of the chloride ions is equal to 0.5 (we cannot in this case consider the total dissociation of MgCl_2_, as this salt is more acidic than NaCl [13]). E°_MgCl2/Mg_ is calculated as −2.69 V/Cl_2_. Therefore,

E_NaMgCl_ = −1.33V/Cl_2_ (18)

The value experimentally determined by Choi et al. [20] was −1.36 V/Cl_2_, which is very close to the theoretical value. Note that the second term of Relations (15) and (17) is generally negligible compared with the first term.

### 4.2. Experimental Salt Redox Potential Measurement

**Figure 5:**
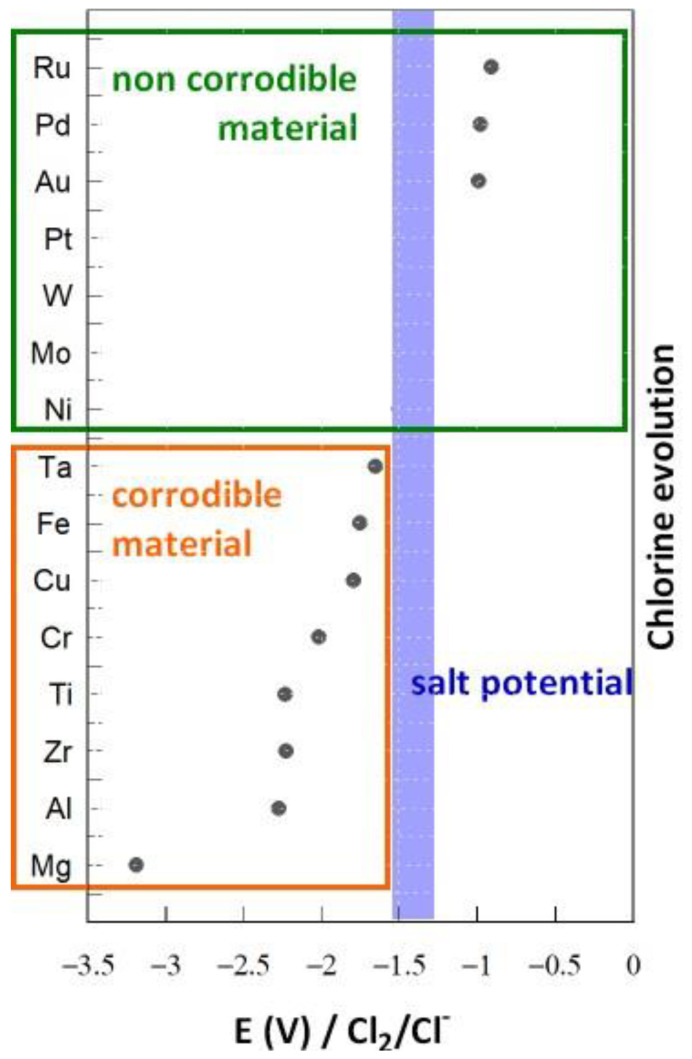
OCP measurements of several electrodes of pure metallic materials in molten LiCl-KCl salt at 500 °C.

Figure 5 presents the open-circuit potentials measured on several pure metallic electrodes in LiCl-KCl at 500 °C. The most effective electrode material for measuring the redox potential of this salt is tungsten, because this element is not oxidized in the electroactivity domain of chloride salts, and so chlorine evolution can be observed [11]. Mo and Ni have potentials close to that of W and can be considered inert in this salt. Elements with a higher potential than that of the salt are not corroded, as they are in their immunity domain. On the contrary, elements whose potential is lower than that of the salt are corroded.

This section presented a methodology for calculating the salt redox potential and measuring it in the molten salt. We observed that Cr and Fe are corroded in the salt if the redox potential is not modified.

## 5. Corrosion Mitigation or Inhibition

To decrease or inhibit corrosion, the redox potential of the salt must be held at a value lower than the Cr and Fe oxidation potential. Therefore, the objective is to achieve a potential close to −2 V/Cl_2_ (see Figure 5). The addition of Mg metal decreases the potential to −1.93 V/Cl_2_ [20]. In these conditions, corrosion decreases substantially, but this option can only be used in a molten salt containing MgCl_2_. Moreover, the amount of Mg required to maintain a low potential for long periods remains to be studied. In this section, three methods for controlling the salt redox potential are proposed.

### 5.1. Addition of a Redox System

**Figure 6:**
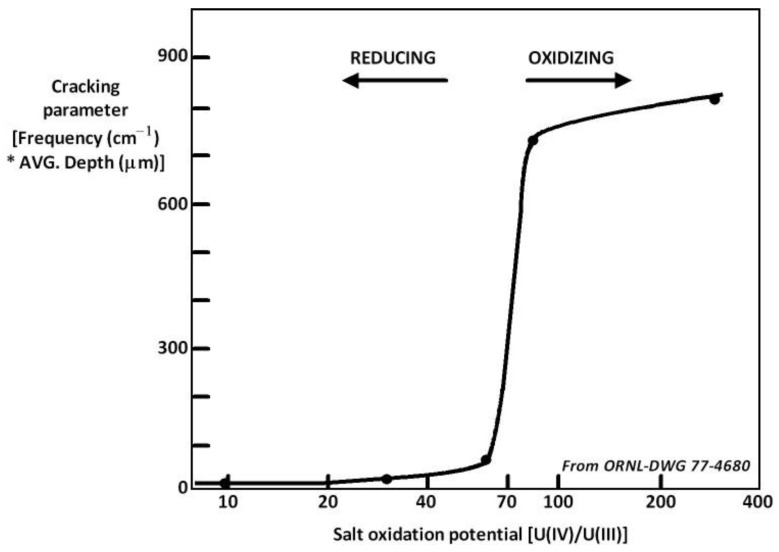
Cracking parameter (depth) as a function of the U(IV)/U(III) ratio LiF-BeF_2_ containing uranium fluoride [27].

The addition of a soluble/soluble redox system is a promising method for controlling the salt redox potential. This technique was tested by the Oak Ridge National Laboratory, USA when they developed the MSRE. They used a fluoride salt (LiF-BeF_2_) containing uranium fluoride and corrosion was a serious issue. To decrease the corrosion, they proposed controlling the redox potential by governing the UF_4_/UF_3_ ratio in the salt. Figure 6 illustrates the influence of the U(IV)/U(III) ratio on corrosion, evaluated according to the thickness of cracks in the material samples [27]. When the U(IV)/U(III) ratio was over 60, the corrosion became significant. These experiments were conducted in the presence of tellurium, which severely corrodes metals by inducing intermetallic compound formation.

**Figure 7:**
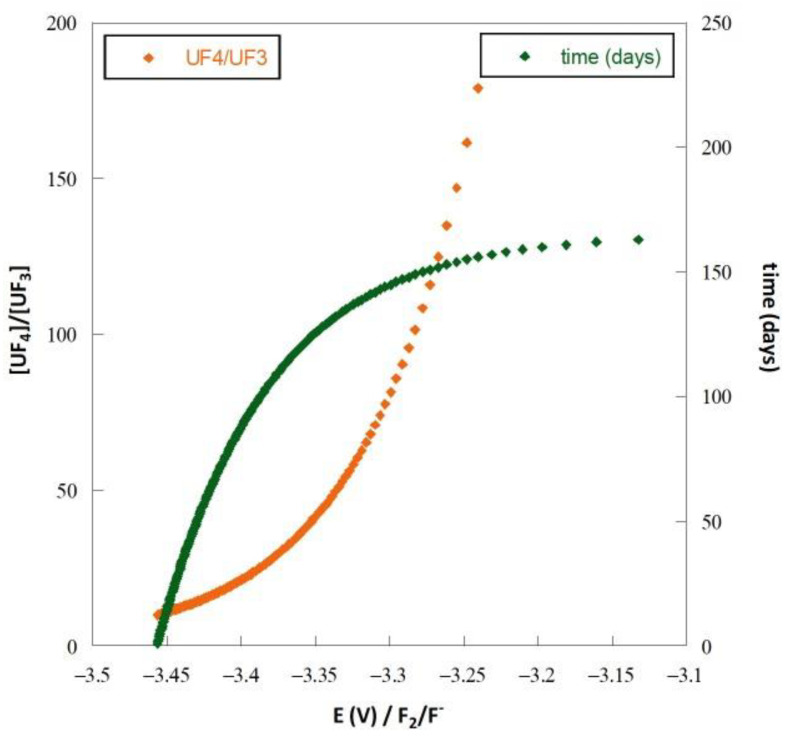
Evolution of the potential according to the U(IV)/U(III) ratio (in red) and the duration of reactor operation (in green) in LiF-ThF_4_ at 600 °C containing uranium.

In fluoride media, as in the case of nuclear applications, controlling the U(IV)/U(III) ratio is the recommended method to avoid corrosion [28,29]. However, during reactor operation, the redox potential increases as the U(IV)/U(III) ratio varies over time (Figure 7). The calculation is made by considering a uranium consumption of 16.2 mole per day, the value determined for the Molten Salt Fast Reactor (MSFR) concept in fluoride salt (LiF-ThF_4_ containing uranium fluoride at 600 °C) by Doligez [30].

The U(IV)/U(III) ratio evolves due to the fission reaction that can occur for both U(IV) and U(III). As presented in [31,32], the fission reaction mainly leads to the formation of gas, metals, or elements with a valence of (III) as follows:

UF_4_ + n → MF_3_ +gas or metal + ½ F_2_ + energy (19)

UF_3_ + n → MF_3_ +gas or metal + energy (20)

UF_3_ + ½ F_2_ → UF_4_ (21)

The fission reaction increases the U(IV)/U(III) ratio. Thus, during operation, the potential must be kept low by adding a reducing element. During MSRE operation, Be metal was added because the salt was the mixture LiF-BeF_2_. In the case of the MSFR, which contains the salt LiF-ThF_4_-UF_4_, the addition of U metal is recommended to decrease the potential during operation. The reaction is

3UF_4_ + U → 4UF_3_ (22)

According to Reaction (22), the theoretical potential reaches an equilibrium between the UF_4_ consumed, UF_3_ produced, and U metal. The following relation assesses the equilibrium potential between these three forms of uranium:

(23) $$E_{UF4/UF3/U}=\frac{3E{}^{\circ}\prime_{UF3/U}+E{}^{\circ}\prime_{UF4/UF3}}{4}+\frac{2.3RT}{4F}\log x(UF_{4})$$

**Figure 8:**
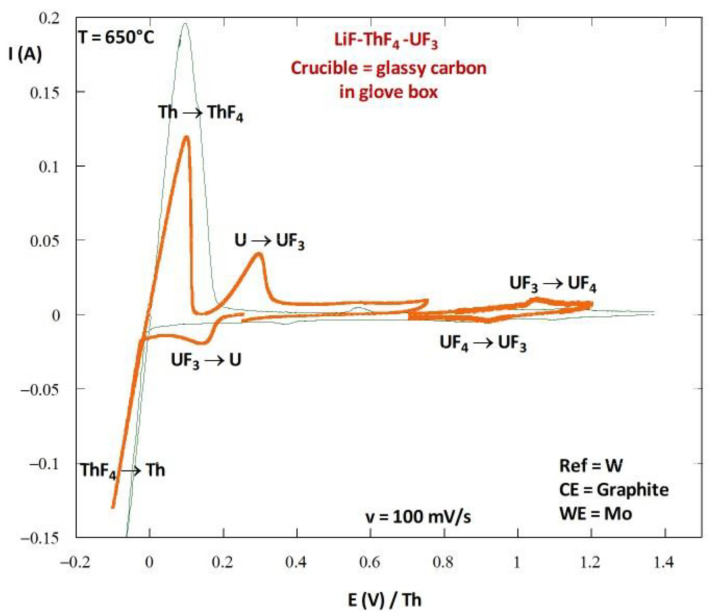
Cyclic voltammogram recorded in LiF-ThF_4_ (75–25 mol%) at 100 mV/s on Mo electrode before (green curve) and after (orange curve) the introduction of UF_3_ by the chemical oxidation of U metal for 24 h in the salt.

Here, E°′_UF3/U_ and E°′_UF4/UF3_ are the apparent potentials (taking account of the solvation properties) of the redox systems UF_3_/U and UF_4_/UF_3_, respectively, which can be determined by cyclic voltammetry (Figure 8), and x(M) represents the mole fraction of M. On the curve in Figure 8, the systems UF_3_/U and UF_4_/UF_3_ can be observed, and the potential values of E°′_UF3/U_ and E°′_UF4/UF3_ are shown to be 0.2 and 0.95 V/Th, respectively. Therefore, the equilibrium potential corresponding to the UF_4_/UF_3_/U system is 0.39 V/Th for a UF_4_ mole fraction of 1 mol%.

Now, we consider a molten LiF-ThF_4_-UF_4_ (77-22-1 mol%) salt. The OCP of an inert electrode (W or Mo) measured in this salt is higher than 1.2 V/Th. After introducing U metal to this salt, the metal’s potential shifts from 0.1 V/Th to 0.42 V/Th, in agreement with the calculated value. After removing the uranium metal, the OCP measured at an inert electrode is close to 0.7 V/Th, which is between the two redox systems of U. The introduction of uranium metal to a molten salt containing UF_4_ seems to effectively decrease the redox potential.

In chloride salts, the standard potential of the redox system UCl_4_/UCl_3_ is higher than that of the Cr and Fe systems. Thus, it is not promising as a redox buffer. Moreover, the vaporization temperature of UCl_4_ is 590 °C, which is too low for it to be used in an MSR to control the fuel salt redox. Another soluble/soluble redox system could be considered, such as the redox couple TiCl_3_/TiCl_2_, which presents a low redox standard potential but must also be assessed from a neutronic point of view.

### 5.2. Potentiostatic Control of the Material Potential

**Figure 9:**
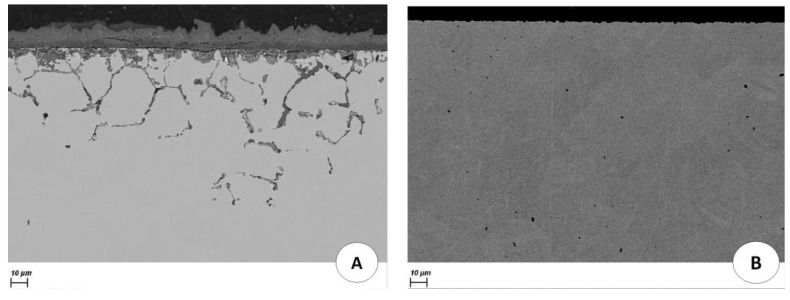
Cross-sectional SEM-BSE images of AISI 304L after 360 h of immersion in molten LiF-LiCl salt at 530 °C without (**A**) and with potential control (**B**).

**Figure 10:**
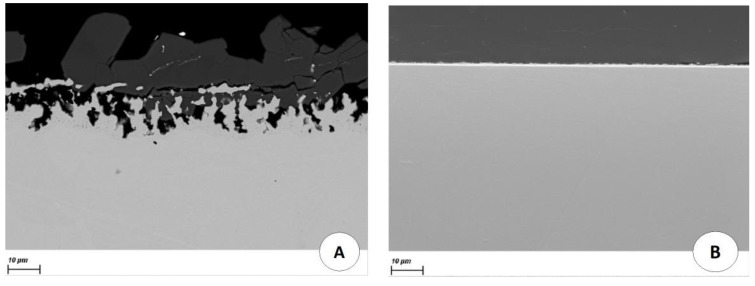
Cross-sectional SEM-BSE images of Hastelloy C276 after 360 h of immersion in molten LiF-LiCl salt at 530 °C without (**A**) and with potential control (**B**).

Another way to control the redox potential is to directly apply a potential to the structural material by an electrical contact with a potentiostat. The corrosion of Hastelloy C276 and AISI 304L stainless steel immersed for two weeks in a molten LiCl-LiF (69.5–30.5 mol%) salt at 530 °C with and without potential control was compared (Figure 9 and Figure 10). The potential applied was −2.3 V/Cl_2_, which is equivalent to a U(IV)/U(III) ratio lower than 10.

The alloys immersed without potential control presented severe corrosion. In contrast, when a potential was applied to the alloys, no corrosion was observed. The materials were in their immunity domain and were not corroded. This experiment proved the role of the potential in the corrosion of these alloys.

Note the importance of applying a potential rather than a current. Indeed, applying a current could lead to the reduction of the solvent, which is not required.

### 5.3. Addition of an Amphoteric Compound

**Figure 11:**
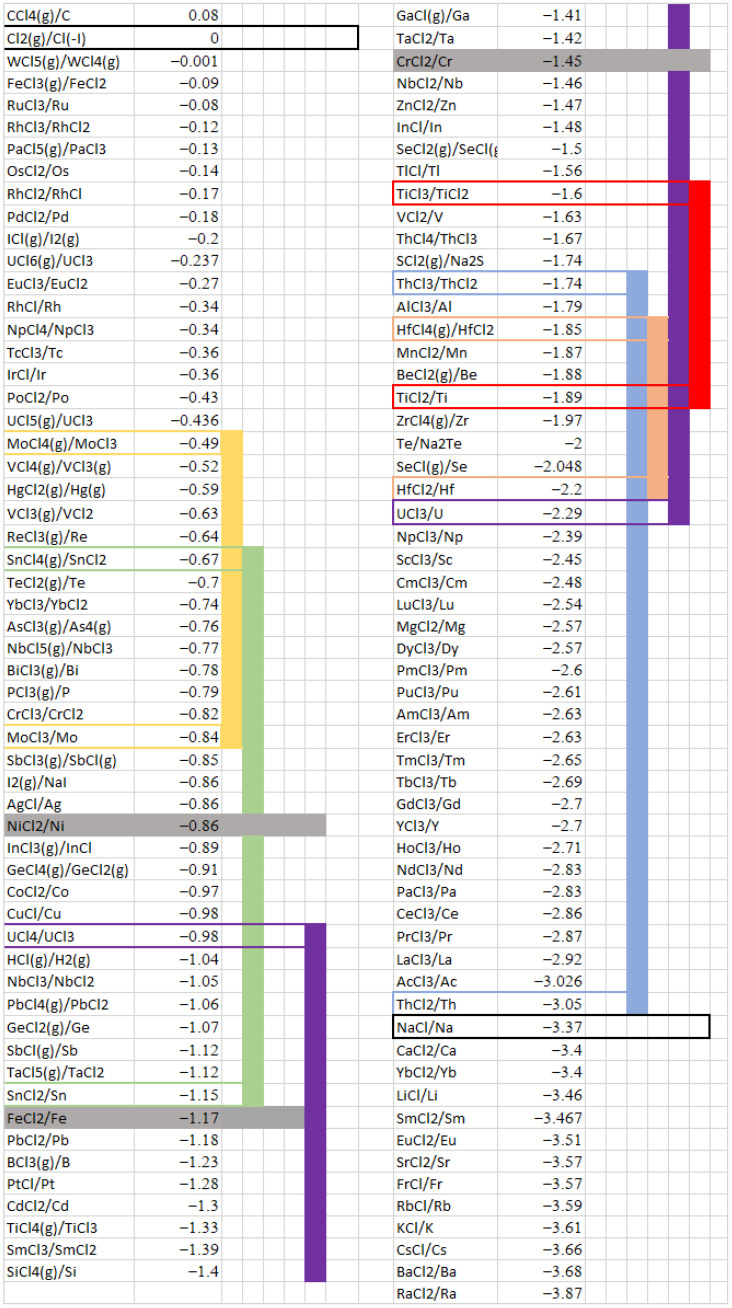
Standard potentials (given against the reference Cl_2_(g)/Cl^−^) of several redox systems and stability domains of amphoteric compounds at 650 °C. Stability domains of several amphoteric compounds: MoCl_3_—yellow, SnCl_2_—green, UCl_3_—purple, TiCl_2_—red, ThCl_2_—light blue, HfCl_2_—orange; Black boxes: reduction in Na and Cl_2_ formation; in grey: standard potentials of Cr, Fe and Ni.

An amphoteric element is both an oxidant and a reductant. Therefore, it constrains the potential of the solvent between the two limits of its oxidation and its reduction. Figure 11 presents the redox standard potentials of several redox systems calculated using the database HSC Chemistry. The colors represent the stability domains of several amphoteric compounds: MoCl_3_—yellow, SnCl_2_—green, UCl_3_—purple, TiCl_2_—red, ThCl_2_—light blue, and HfCl_2_—orange. The limits of the solvent, i.e., the reduction in Na and Cl_2_ formation, are indicated in black boxes. We highlight in gray the standard potentials of Cr, Fe, and Ni. The colored domains correspond to the stability domains of certain soluble compounds. For example, MoCl_3_ and SnCl_2_ present a stability domain at a higher potential than Fe and Cr. Therefore, the addition of SnCl_2_ or MoCl_3_ constrains the redox potential of the salt within a domain corresponding to the oxidation of the alloys. In contrast, ThCl_2_, TiCl_2_, and HfCl_2_ are stable in a potential domain lower than that for Cr oxidation. However, the existence of ThCl_2_ is controversial. Some authors have observed a reaction between Th metal and ThCl_4_ to form ThCl_2_ [32,33]. Others have demonstrated the direct oxidation of Th metal to ThCl_4_ [34,35]. These conflicting studies indicate the partial stability of ThCl_2_, showing that it is not a promising candidate for stabilizing the redox potential.

UCl_3_ is an important compound for nuclear reactor application because it is fissile and/or fertile and, consequently, is generally present in the fuel salt. This element presents a large stability domain, which includes the systems of Cr and Fe. Using the methodology described in Section 4.1, one can assess the redox potential of a salt containing UCl_3_ by considering the two redox reactions limiting its stability domain:

UCl_3_ + Cl^−^ → UCl_4_ +e (24)

UCl_3_ + 3e → U + 3Cl^−^ (25)

We deduce the following relation:

(26) $$E_{UCl3}=\frac{E{{}^{\circ}}_{UCl4/UCl3}+3E{{}^{\circ}}_{UCl3/U}}{4}+\frac{2.3RT}{F}\log\frac{3}{a{\left(Cl^{-}\right)}^{4}}$$

**Figure 12:**
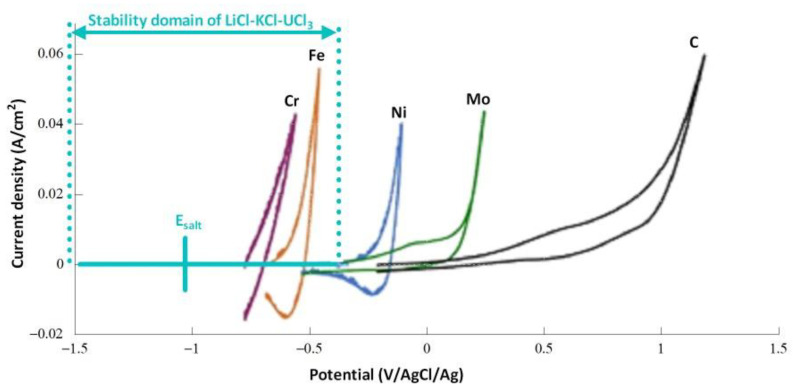
Stability domain of LiCl-KCl-UCl_3_ experimentally measured by Hoover et al. [36] and the voltammograms recorded in LiCl-KCl on several working electrodes at 100mV/s at 500 °C.

The equilibrium potential calculated using Relation (26), considering the standard potentials of Figure 11 and a chloride ion activity of 1, is −1.88V/Cl_2_. As this potential is lower than the standard potentials of the Cr and Fe systems, we expect that these elements are in their immunity domain in the presence of UCl_3_. Hoover et al. [36] reported the behavior of UCl_3_ in a eutectic LiCl-KCl mixture at 500 °C. The OCP measured for the salt was close to −2.03 V/Cl_2_ (−1 V/(AgCl/Ag)). The difference between the experimental and theoretical potentials can be attributed to the solvation of U(III). These experimental data are very encouraging, as they demonstrate that the redox potential of the salt can be mitigated by introducing an amphoteric element. Figure 12 presents a comparison of the stability domain determined by Hoover et al. and the voltammograms recorded on several working electrodes in LiCl-KCl at 500 °C. We observed the oxidation of Cr and Fe at a potential higher than the redox potential of the salt containing UCl_3_. Corrosion tests of austenitic steels were conducted in NaCl-KCl salts containing UCl_3_ at 750 °C [37]. Corrosion was evidenced with the dissolution of mainly Cr, Fe, and Mn, and the corrosion increased with the uranium concentration. These results do not agree with our expectations, but another parameter must be considered: the authors observed that the corrosion followed an electrochemical process and was more substantial in the solvent containing U^4+^ ions. The presence of U^4+^, which is an oxidizing impurity, modified the chemistry and redox potential of the salt. Considering Figure 11 and the redox potential of the system U(IV)/U(III), corrosion in an electrolyte containing U^4+^ seems reasonable. The presence of impurities is a key problem for controlling the corrosion of materials, and caution should be exercised before drawing conclusions.

Based on the thermodynamic approach and experimental determination of chloride salt redox potentials, UCl_3_ is a very promising candidate for controlling the redox potential of molten salts and mitigating the corrosion of structural materials. However, uranium is not suitable for all applications, and other options can be considered.

Figure 11 indicates that other amphoteric elements could be considered, in particular HfCl_2_ and TiCl_2_.

Hafnium was studied in a NaCl-KCl salt by Kusnetsov [38]. The two redox systems, HfCl_2_/Hf and HfCl_4_/HfCl_2_, were observed at −2.2 V/Cl_2_ and −1.6 V/Cl_2_, respectively, in agreement with the thermodynamic values based on the solvation effect of the chloride salt.

Titanium has been extensively studied in molten chloride salts [39,40,41,42]. Martinez et al. [40] determined that the apparent standard redox potentials of the two systems Ti(III)/Ti(II) and Ti(II)/Ti(0) in the molten chloride salt NaCl-CaCl_2_ are −1.945 and −2.013 V/Cl_2_, respectively. These values are close to those determined by Ferry [41] in LiCl-KCl at 470 °C: −1.938 V and −2.053 V/Cl_2_, respectively. This standard potential value for Ti(III)/Ti(II) is lower than that calculated using the HSC Chemistry database. This is probably because the solvation power of the solvent is much stronger for Ti(III) compared with Ti(II). This has been observed with valencies (III) and (II) of americium: the solvation of Am(III) is 10 times higher than that of Am(II) [43].

HfCl_2_ and TiCl_2_ present potential stability domains lower than the oxidation potentials of Cr, Fe, and Ni, suggesting that they could be used as additives in molten chloride salts to control the redox potential. However, hafnium cannot be added to nuclear reactor fuel because of its neutronic poison properties. Moreover, the price of this element in HfCl_4_ form is very high. Titanium could be effective for limiting corrosion in molten salt reactor coolants and other applications, notably solar energy storage, which requires slightly corrosive molten chloride salts.

Experiments were performed by adding Ti(II)/Ti(III) to LiCl-KCl through the oxidation of Ti metal. A total of 0.015 moles of Ti was oxidized, corresponding to 0.8 mol% in the salt. The redox potential of the salt after more than 1 month was measured as −2.13 V/Cl_2_, and no variation was recorded over time. This potential corresponds to the immunity domain of Cr and Fe (Figure 5). By considering the following Nernst relations and the total amount of titanium introduced into the salt, the amounts of Ti(III) and Ti(II) in the salt can be determined.

(27) $$E_{salt}=E{{}^{\circ}}_{Ti(III)/Ti(II)}+\frac{2.3RT}{F}\log\frac{x(Ti(III))}{x(Ti(II))}$$

(28) $$E_{salt}=E{{}^{\circ}}_{Ti(II)/Ti}+\frac{2.3RT}{2F}\log x(Ti(II))$$

In these relations, x(M) is the mole fraction of M, the activity coefficients of Ti(II) and Ti(III) are 1, and the activity of Ti metal is 1. If the Ti metal is oxidized only to Ti(II), considering Relation (28) and the apparent redox potentials determined by Ferry [41], the potential should be −2.37V. We can conclude that the Ti metal is oxidized to Ti(II) and Ti(III). From Relation (27), we determine

x(Ti(III)) = 4.7.10^−4^ and x(Ti(II)) = 7.5.10^−3^ (29)

Considering the concentration of Ti(II) and Relation (28), the potential of the salt is −2.2 V/Cl_2_, in agreement with the experimental measurement of −2.13 V/Cl_2_.

**Figure 13:**
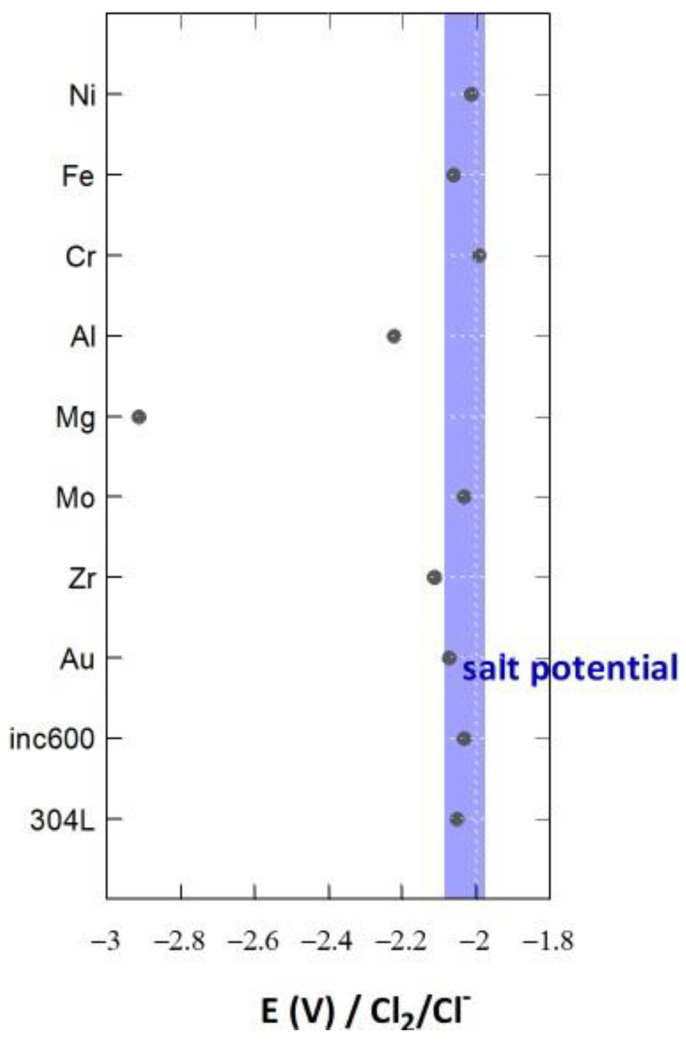
OCP measured on several electrodes of pure metallic materials in molten LiCl-KCl-TiCl_2_-TiCl_3_ salt at 500 °C.

Several electrodes were introduced into the molten salt containing Ti(III) and Ti(II), and their OCPs were measured (Figure 13). Most of the metals presented an OCP corresponding to the salt redox potential. Thus, they presented inert electrode behavior and were not corroded in these conditions. Three metals presented a lower potential: Al, Zr, and Mg. Zr and Al had standard potentials close to that of the redox system Ti(II)/Ti (Figure 11), and Mg had a very low potential compared with Ti. Mg would be severely corroded in a salt containing Ti(III)/Ti(II), Zr and Al would present slight corrosion, and the corrosion of the other materials would be inhibited by the presence of titanium.

**Figure 14:**
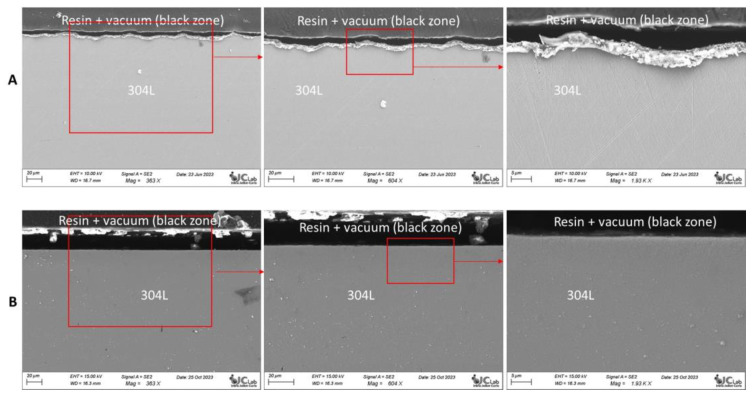
Cross-sectional SEM-SE images of 304L immersed for 1 week in LiCl-KCl at 500 °C (successive zooms from left to right): (**A**) Without addition of Ti. (**B**) With addition of Ti.

We immersed 304L stainless steel samples in molten LiCl-KCl salt for one week. The SEM analysis of cross-sections of the samples immersed in the molten salt with and without Ti is presented in Figure 14. The samples immersed in the molten salt containing Ti presented no corrosion. On the contrary, corrosion was observed when the alloys were immersed in molten salt without Ti. This confirmed the influence of the Ti(III)/Ti(II) system on the redox potential of the molten salt, as well as the influence of the salt’s redox potential on the corrosion of materials. The corrosion mitigation by the redox control of the salt has also been demonstrated in the case of titanium in molten chlorides in a work performed by CEA/Marcoule and Orano, a patent was published at the end of 2023.

The amount of soluble titanium that is required to constrain the salt’s redox potential with time has to be optimized both for nuclear reactors and for other applications. In the case of molten salt reactors, the fission reaction leads to an increase in the potential. In this case, the addition of the amphoteric element must be carried out regularly so as to counterbalance the increase in potential. In the case of another application, if no introduction of an oxidizing element is planned over time, it is estimated that 1% of an amphoteric element makes it possible to regulate the salt’s redox potential. However, long-term experiments are planned in order to optimize the quantity of amphoteric element to be introduced into the salt.

## 6. Conclusions

Corrosion occurs in molten salts when the redox potential of the salt is higher than the standard potentials of the Cr and Fe systems. Thermodynamic calculations demonstrated that the alloys’ corrosion inhibition can be reached if the redox potential of the salt is kept in the immunity domain of the elements. These calculations also showed that the lower the oxo-acidity, the smaller the redox potential corresponding to the immunity domain of the metallic element and, therefore, the more difficult it is to achieve.

A methodology was proposed to determine the theoretical salt’s redox potential, and a tungsten electrode was chosen as the working electrode to experimentally measure this value in chloride media.

Several options for mitigating corrosion were proposed in this paper. The addition of a redox system, the application of a cathodic potential, and the use of amphoteric species are three options for controlling the redox potential of a molten salt and limiting its corrosiveness. Immersion tests were performed to show the efficiency of redox potential control in mitigating the corrosion of metallic samples. In chloride media, the use of UCl_3_ and TiCl_2_ seems an effective way to control the redox potential of the salt. To complete this study, experiments will be carried out in order to establish the relationship between the quantity of amphoteric species added and the stabilization of the redox potential as a function of time to optimize the quantity of amphoteric element required to stabilize the salt’s redox potential. Molten salt containing UCl_3_ will be studied to verify the influence of UCl_3_ on material corrosion. We plan to study the evolution over time of the salt’s redox potential containing 1 mol% UCl_3_ and to perform immersion tests in order to confirm whether the metal coupons are corroded or not.

## Data Availability

Data are contained within the article.
